# Development of a Sustained Release Nano-In-Gel Delivery System for the Chemotactic and Angiogenic Growth Factor Stromal-Derived Factor 1α

**DOI:** 10.3390/pharmaceutics12060513

**Published:** 2020-06-04

**Authors:** Joanne O’Dwyer, Megan Cullen, Sarinj Fattah, Robert Murphy, Smiljana Stefanovic, Lenka Kovarova, Martin Pravda, Vladimir Velebny, Andreas Heise, Garry P. Duffy, Sally Ann Cryan

**Affiliations:** 1 Drug Delivery & Advanced Materials Team, School of Pharmacy & Biomolecular Sciences, Royal College of Surgeons in Ireland (RCSI), Dublin 2, Ireland; joanneodwyer@rcsi.ie (J.O.); mecullen@tcd.ie (M.C.); sarinjfattah@rcsi.ie (S.F.); smiljanastefanovic@rcsi.ie (S.S.); 2 Tissue Engineering Research Group, Department of Anatomy & Regenerative Medicine, Royal College of Surgeons in Ireland (RCSI), Dublin 2, Ireland; 3 Trinity Centre for Biomedical Engineering, Trinity College Dublin (TCD), Dublin 2, Ireland; garry.duffy@nuigalway.ie; 4 SFI Research Centre for Medical Devices (CURAM), National University of Ireland Galway (NUIG) & Royal College of Surgeons in Ireland (RCSI), Galway and Dublin, Ireland; andreasheise@rcsi.ie; 5 Department of Chemistry, Royal College of Surgeons in Ireland (RCSI), Dublin 2, Ireland; robertdmurphy@rcsi.ie; 6 R & D Department, Contipro, Dolni Dobrouc 401, 561 02 Dolni Dobrouc, Czech Republic; lenka.kovarova@contipro.com (L.K.); martin.pravda@contipro.com (M.P.); Vladimir.Velebny@contipro.com (V.V.); 7 Faculty of Chemistry, Institute of Physical Chemistry, Brno University of Technology, Purkynova 464/118, 612 00 Brno, Czech Republic; 8 The SFI Centre for Advanced Materials and Bioengineering Research (AMBER), National University of Ireland Galway (NUIG), Royal College of Surgeons in Ireland (RCSI) & Trinity College Dublin (TCD), Dublin, Ireland; 9 Anatomy, School of Medicine, College of Medicine, Nursing and Health Sciences, National University of Ireland Galway (NUIG), Galway, Ireland

**Keywords:** angiogenesis, stromal-derived factor, sustained release, nanoparticle, hydrogel, chemotaxis, protein delivery

## Abstract

Stromal-Derived Factor 1α (SDF) is an angiogenic, chemotactic protein with significant potential for applications in a range of clinical areas, including wound healing, myocardial infarction and orthopaedic regenerative approaches. The 26-min in vivo half-life of SDF, however, has limited its clinical translation to date. In this study, we investigate the use of star-shaped or linear poly(glutamic acid) (PGA) polypeptides to produce PGA–SDF nanoparticles, which can be incorporated into a tyramine-modified hyaluronic acid hydrogel (HA–TA) to facilitate sustained localised delivery of SDF. The physicochemical properties and biocompatibility of the PGA–SDF nanoparticle formulations were extensively characterised prior to incorporation into a HA–TA hydrogel. The biological activity of the SDF released from the nano-in-gel system was determined on Matrigel^®^, scratch and Transwell^®^ migration assays. Both star-shaped and linear PGA facilitated SDF nanoparticle formation with particle sizes from 255–305 nm and almost complete SDF complexation. Star-PGA–SDF demonstrated superior biocompatibility and was incorporated into a HA–TA gel, which facilitated sustained SDF release for up to 35 days in vitro. Released SDF significantly improved gap closure on a scratch assay, produced a 2.8-fold increase in HUVEC Transwell^®^ migration and a 1.5-fold increase in total tubule length on a Matrigel^®^ assay at 12 h compared to untreated cells. Overall, we present a novel platform system for the sustained delivery of bioactive SDF from a nano-in-gel system which could be adapted for a range of biomedical applications.

## 1. Introduction

Stromal-Derived Factor 1 (SDF) (sometimes referred to as CXCL12) is an 8 kDa chemokine involved in inflammation, hematopoesis and angiogenesis [1]. Two isoforms of SDF exist, SDF -α and SDF -1β, formed from variations in splicing. SDF-1α is the most predominant of the two and is found in almost all organs and all references in this paper to SDF will refer to the α isoform. SDF exerts its action by binding to either the CXCR4 or CXCR7 receptors [2]. CXCR4 is a G-protein-coupled receptor and, upon SDF binding, several intracellular pathways can be activated [3]. SDF can promote cell proliferation and migration and can increase the recruitment of stem cells [4,5]. Furthermore, SDF has been shown to reduce apoptosis and improve the survival of cardiomyocytes following myocardial infarction (MI), leading to enhanced heart function and overall survival [5,6,7]. It has also been suggested that SDF may prevent reperfusion injury following an ischaemic event [8]. More recently, SDF has been shown to improve microvessel formation in vitro and angiogenesis in vivo due to its interaction with the CXCR7 receptor [3,9].

The various biological actions of SDF mean that it could be beneficial in a number of biomedical applications. For example, angiogenesis is desired in the aftermath of MI to improve blood supply to cells in the ischaemic area [10,11]. SDF can prevent reperfusion injury and reduce infarct size when administered following the development of myocardial ischaemia [8]. Angiogenesis is also important in wound healing and SDF-induced angiogenesis has improved vascularization in full thickness skin wounds in mice, improving overall wound healing time [12]. Improved vascularization could also be useful for treatment of critical limb ischaemia [13]. Other potential applications of SDF include repair of damaged peripheral neurons, stem cell recruitment following traumatic brain injury and enhanced bone repair due to stem cell migration into the bone defect site [14,15,16]. In recent years, tissue engineering approaches have received much attention for the regeneration or repair of damaged tissues [17]. Tissue engineering commonly employs scaffolds to guide tissue repair and attraction of stem cells to these scaffolds and their vascularization has been identified as a major factor which will determine the success of these tissue engineering strategies [18,19,20]. Chemokines, such as SDF, are proposed to be essential for this process of scaffold integration and this has been shown by the inclusion of SDF in a poly(caprolactone) scaffold, which improved migration of mesenchymal stem cells into the scaffold in a rodent model of bone repair [21,22,23,24]. In rabbits, collagen scaffolds loaded with SDF improved chondrogenesis, potentially due to an increase in stem cell recruitment into the scaffold [25]. Therefore, SDF could have a significant impact on the treatment of several medical conditions and in the development of effective regenerative therapies if presented and delivered appropriately.

The clinical translation of SDF-containing therapies has so far been limited due to the short half-life of SDF in vivo of just 26-min, caused by the degradation of the protein by matrix metalloproteinases, dipeptidylpeptidases, neutrophil elastase and cathepsin G [26,27]. This means that either SDF is degraded by proteases before it can bind to its receptor, or that achieving therapeutically adequate concentrations of SDF at the target site for a prolonged period is challenging [28]. General strategies for overcoming the short half-life of therapeutic compounds include continuous intravenous infusion, frequent administration or the development of sustained or prolonged release formulations [15,29,30]. The sustained release approach offers a range of benefits for the healthcare provider and the patient in terms of therapeutic efficiency, administration and convenience. Therefore, in this work we focused on the development of a novel sustained release system for SDF.

A number of particulate systems have previously been investigated to achieve prolonged release of SDF, but none have been translated to the clinic. SDF nanoparticles reported in the literature to-date use polymers such as poly(lactic-*co*-glycolic acid) (PLGA), dextran or chitosan as protein carriers [12,14,15,16,30,31,32]. The formulation of these nanoparticles can require complex processes such as double-emulsion solvent evaporation or spray-drying. Furthermore, some formulation procedures damage the protein during fabrication and poor SDF encapsulation efficiencies and protein loading have also been reported [14,15,31,33]. In this work, novel polypeptide-based nanocarriers were chosen, as they can be tailored for specific therapeutic cargoes to facilitate an easy self-assembly fabrication approach for drug loading, thereby enabling high protein encapsulation efficiencies and loading capacities [34].

Polypeptide-based nanocarriers have previously been reported in the literature for drug delivery applications including the use of star-shaped poly(lysine) polypeptides to fabricate insulin nanoparticles that supported sustained insulin release [35,36,37]. However, poly(lysine) polypeptides are not suitable for this application as they are positively charged and so would not bind to the positively charged heparin-binding site on SDF. Thus, negatively charged poly(glutamic acid) (PGA) polypeptides are used herein. These linear PGA (L-PGA) and star-shaped PGA (star-PGA) polypeptides are analogous to those previously synthesized by Byrne et al. [38]. It is proposed that the negative charge on the PGA will facilitate electrostatic interaction with the positively charged heparin-binding site on the SDF [39]. Interaction with this heparin-binding site has been reported to protect SDF from degradation by proteases in vivo without affecting the ability of SDF to interact with its receptor and exert its therapeutic effect [32]. L-PGA and star-PGA are investigated herein for their ability to form L-PGA–SDF and star-PGA–SDF nanoparticles.

Rapid clearance from the bloodstream can limit the potential therapeutic effects of nanoparticles and their loaded cargo [40]. Nano-in-gel approaches are a growing area of interest in the field of drug delivery to facilitate the transport of nanoparticles to the desired anatomical/disease site and maintain them at this site [41]. Nano-in-gel systems can be injected or implanted at the site of action, offering the possibility of targeted delivery [41,42]. The double matrix system also facilitates sustained release of the loaded therapeutic. Incorporating SDF nanoparticles in a second matrix, such as a hydrogel, could therefore enable the effective delivery and further aid the sustained release of SDF. In this work we will investigate the incorporation of SDF nanoparticles into a tyramine-modified hyaluronic acid (HA–TA) hydrogel. HA–TA forms a hydrogel rapidly on interaction of the appropriate chemical crosslinkers. A specialized double syringe injection system is required for formulation of the HA–TA hydrogel. This double syringe system prevents premature gelation which would occur if both crosslinkers were in the same syringe. HA–TA hydrogels have been reported as potential candidates for drug delivery [43,44,45]. Intra-articular injection of a dexamethasone-loaded HA–TA hydrogel has been used for treatment of rheumatoid arthritis in rats while, in vitro, alpha-amylase has been loaded into and released from a HA–TA hydrogel [43,44]. Dolan et al. have recently developed a catheter capable of percutaneous endocardial delivery of a HA gel similar to the one used in this paper [43,46]. This Advanced Material Catheter (AMCath) was used to inject a HA–TA hydrogel pre-clinically into a pig heart [46]. AMCath would potentially be able to deliver the PGA–SDF–HA–TA formulation developed herein to the ventricle wall for the promotion of angiogenesis in the heart following an ischaemic event, one of the potential biomedical applications of SDF outlined above.

Herein, we will investigate the potential of PGA polypeptides to form PGA–SDF nanoparticles. Following this, incorporation of the lead SDF nanoparticles into the HA–TA hydrogel will be investigated and the biocompatibility and bioactivity of the resulting nano-in-gel formulation will be assessed using relevant in vitro bioassays.

## 2. Materials and Methods

### 2.1. Materials

Recombinant Human SDF and the SDF ELISA kits used were obtained from R&D Systems (Abingdon, UK). Illustra MicroSpin S-400 HR columns were purchased from GE Healthcare (Amersham, UK). Float-A-Lyzers were obtained from Spectrum Labs (Amsterdam, The Netherlands). EndoGrow cell culture medium was purchased from Merck Millipore Ltd. (Cork, Ireland). Growth factor reduced Matrigel^®^ was purchased from Corning BV (Amsterdam, The Netherlands). Human umbilical vein endothelial cells (HUVECs) were obtained from Lonza Ltd. (Slough, UK). All other chemicals and reagents were sourced from Sigma Aldrich (Dublin, Ireland).

**Figure 1:**
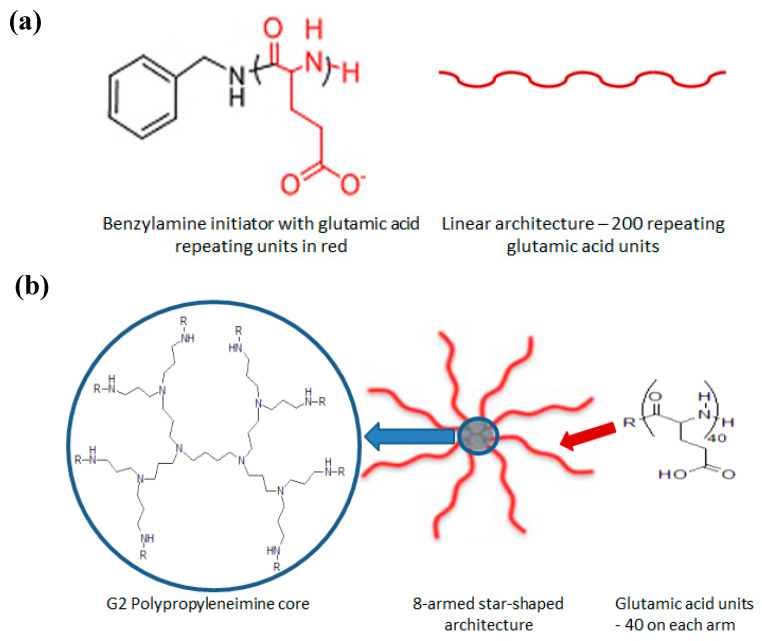
(**a**) The linear poly(glutamic acid) (L-PGA) and (**b**) star-poly(glutamic acid) (star-PGA) polypeptides synthesized, which will be used for nanoparticle fabrication in this work.

L-PGA and Star-PGA were synthesized by Dr Robert Murphy using the method previously outlined by Byrne et al. [38]. The L-PGA, shown in Figure 1a contained 200 glutamic acid residues per molecule (Mn: 26 kDa). The star-PGA, shown in Figure 1b, had a polypropyleneimine (PPI) core and eight arms, each with 40 glutamic acid residues (theoretical Mn: 42 kDa; estimated isoelectric point: 4.1). Gel permeation chromatography was used to ensure polymer formation. The chromatograms obtained were analogous to those previously reported by our group [47].

### 2.2. Nanoparticle Fabrication

Particle dispersions were formed by adding star-PGA or L-PGA to an eppendorf tube containing Phosphate-Buffered Saline (PBS) followed by the addition of the requisite amount of SDF to a final volume of 50 µL. The amount of star-PGA or L-PGA required was calculated based on the desired molar ratio of star-PGA:SDF or L-PGA:SDF. Molar ratios were based on a star-PGA molecular weight of 42 kDa, L-PGA molecular weight of 26 kDa and an SDF molecular weight of 8 kDa. In all initial formulations 25 ng SDF was used from a 1 µg/1 mL SDF stock solution. Three molar ratios of L-PGA:SDF and star-PGA:SDF were trialed, namely 30:1, 40:1 and 50:1. These molar ratios were chosen based on previous work by our research group with PGA-Vascular Endothelial Growth Factor (VEGF) nanoparticles where molar ratios of 30:1, 40:1 and 50:1 produced nano-sized particles. Dispersions were left at room temperature for five minutes, before any further manipulations, to allow complexation to occur.

### 2.3. Nanoparticle Size and Zeta Potential

The Z-average size of the L-PGA–SDF and star-PGA–SDF formulations was investigated using Dynamic Light Scattering (DLS). Preparations were made as described above, to a volume of 50 µL. Following complexation for five minutes at room temperature, molecular-grade water was added to a final volume of 1 mL and the resulting dispersion was placed in a Zetasizer Nano ZS (Malvern Instruments, Malvern, UK). Particle size was determined using a 100 mW laser beam at a backscatter angle of 173°. All measurements were performed at 25 °C and samples were allowed to equilibrate in the machine for two minutes before measurement. The Polydispersity Index (PDI) for each of the samples was recorded to assess the particle size distribution. The zeta potential of the nanoparticles was then assessed on a Zetasizer Nano ZS, using the same preparation procedure as the size measurement.

### 2.4. Nanoparticle Tracking Analysis

Nanoparticle tracking analysis (NTA) was used to further evaluate particle size. NTA was performed on a Nanosight NS 300 (Malvern Instruments, Malvern, UK). Dispersions were made to a 50 µL volume, left for 5 min, then diluted with molecular grade water to 1 mL for analysis. The sample was then injected into a flow-through cell using the automated injection system on the machine. Thermoelectric Peltier elements on the machine allowed for temperature control at 22 °C during sample measurement. Real-time images were obtained for sixty seconds and this ‘video’ was then analyzed using a suitable particle detection threshold.

### 2.5. Transmission Electron Microscopy

The morphology and size of the star-PGA–SDF 30:1, 40:1 and 50:1 nanoparticles was investigated using Transmission Electron Microscopy (TEM). Nanoparticle dispersions were formulated as per Section 2.2 above in nuclease free water. A 10 µL volume of each dispersion was dropped on to separate silicon monoxide/Formvar^®^ coated copper grids. The grids were left overnight to dry prior to imaging. Imaging was performed on a FEI Tecnai 120 microscope, operating at 80 kV. Nanoparticle size was measured using Image J software Version 1.51.

### 2.6. Nanoparticle Complexation Efficiency and Loading Content

Illustra MicroSpin S-400 HR columns were used to determine the amount of SDF complexed to the PGA, with intact PGA–SDF nanoparticles retained on the column and non-encapsulated SDF eluted. Initial optimization work was performed to determine the amount of free SDF that might be retained in the column and the time required for elution of free SDF. Columns were prepared following the manufacturer’s instructions and 100 µL of each of the nanoparticle dispersions was added to the spin column, followed by centrifugation in a VWR Galaxy 14D microcentrifuge at 700× *g* for two minutes. The eluted liquid was removed and stored at −80 °C for later analysis using an Enzyme-Linked Immunosorbent Assay (ELISA). Complexation efficiency was calculated using the following equation:

Complexation Efficiency (%) =

$$\frac{Amount\;of\;SDF\;originally\;loaded\;\left(ng\right)-SDF\;eluted\;from\;spin\;column\;\left(ng\right)\;}{Amount\;of\;SDF\;originally\;loaded\;\left(ng\right)}\times \frac{100}{1}$$

The loading content of the PGA–SDF nanoparticles was calculated, taking into account the complexation efficiency. The loading content was calculated using the following equation [48]: Loading Content (%) =

$$\frac{Amount\;of\;SDF\;encapsulated\;\left(ng\right)}{Initial\;mass\;of\;the\;nanoparticles\;\left(ng\right)\;}\times \frac{100}{1}$$

### 2.7. In Vitro Biocomtapibility Testing of L-PGA–SDF and Star-PGA–SDF Nanoparticles

#### 2.7.1. Culture of Human Umbilical Vein Endothelial Cells

Human Umbilical Vein Endothelial Cells (HUVECs) were used for all biocompatibility and bioactivity testing. HUVECs represent a good test for biocompatibility as they are the first cells encountered by formulations delivered intravenously. HUVECs are also involved in angiogenesis, one of the biological effects of SDF [9,21,49]. Following revival, HUVECs were cultured at 37 °C and 5% CO_2_. HUVECs were seeded at a density of 1 × 10^6^ cells in T75 flasks and were passaged when they reached 80% confluency. HUVECs were fed with EndoGrow medium containing all required growth supplements (rhVEGF 5 ng/mL, rhEGF 5 ng/mL, rhFGF 5 ng/mL, rhIGF-1 15 ng/mL, ascorbic acid 50 µg/mL, hydrocortisone hemisuccinate 1 µg/mL, heparin sulphate 0.75 U/mL, L-glutamine 10 mM, Foetal Bovine Serum (FBS) 2% and penicillin/streptomycin 1%). VEGF was used at a concentration of 5 ng/mL for feeding the cells during expansion, but was removed in all cases for the assay process to avoid interference with the effects of the SDF treatments.

#### 2.7.2. Biocompatibility of L-PGA–SDF and Star-PGA–SDF

The biocompatibility of the newly formulated nanoparticles was tested to investigate their suitability for biomedical applications. Live/Dead Cell Viability Staining was used to examine the biocompatibility of the formed nanoparticles. HUVECs at P4 were seeded in a 24-well Corning^®^ Costar^®^ tissue culture plate at a seeding density of 3 × 10^4^ cells per well. Cells were given fully supplemented endothelial medium for 24 h. This medium was then removed and replaced with medium containing all supplements except VEGF. HUVECs in the ‘cells alone’ group received only medium. Treatment groups included non-encapsulated SDF or L-PGA–SDF or star-PGA–SDF nanoparticles all at a concentration of 25 ng/mL SDF, based on the SDF dose previously shown to reduce ischaemia/reperfusion injury ex vivo in mouse hearts [50]. Based on the physicochemical characterization data and the principle of bracketing (using extremes of design factors, in this case the highest and lowest PGA doses) used in the pharmaceutical industry the nanoparticle formulations used were L-PGA–SDF 30:1 and 50:1 and star-PGA–SDF 30:1 and 50:1 [51]. At the selected time points: 24, 48 and 72 h, the medium was removed and the cells were washed three times with PBS. A 100 μL volume of Live/Dead solution (2 μM calcein AM, 4 μM ethidium homodimer) was then added to the well and left to incubate for 15 min protected from light at room temperature. Wells were imaged using a fluorescent microscope (Leica Microsystems, Heerbrugg, Switzerland) and Live and Dead images were merged using ImageJ software.

As a further assessment of the biocompatibility of the L-PGA–SDF and star-PGA–SDF nanoparticles, metabolic activity of HUVECs exposed to L-PGA–SDF 30:1, L-PGA–SDF 50:1, star-PGA–SDF 30:1 and star-PGA–SDF 50:1 was measured. As for the Live/Dead assay, 3 × 10^4^ HUVECs were seeded in each well of a 24-well plate. HUVECs were given 1 mL of fully supplemented EndoGrow cell culture medium for the first 24 h. This was then removed and treatments, L-PGA–SDF 30:1, L-PGA–SDF 50:1, star-PGA–SDF 30:1 and star-PGA–SDF 50:1 were added. Non-encapsulated SDF at a concentration of 25 ng/mL was used as the positive control as this matched the SDF dose in all the nanoparticle treatments. HUVECs in EndoGrow medium without SDF and VEGF were used as a control. At the selected time points of 24, 48 and 72 h, medium was removed and 100 μL of CellTiter 96^®^ Aqueous One Solution Cell Proliferation Assay (MTS) and 500 µL EndoGrow (without VEGF or SDF) was added to each well. The plate was incubated at 37 °C for three hours. Absorbance was measured on a Varioskan plate reader at 490 nm. The metabolic activity in each of the treatment groups was normalized to that of the cells alone (untreated) group at each time point.

### 2.8. Preparation of Star-PGA–SDF Nanoparticle-Loaded Hyaluronic Acid Hydrogels

**Figure 2:**
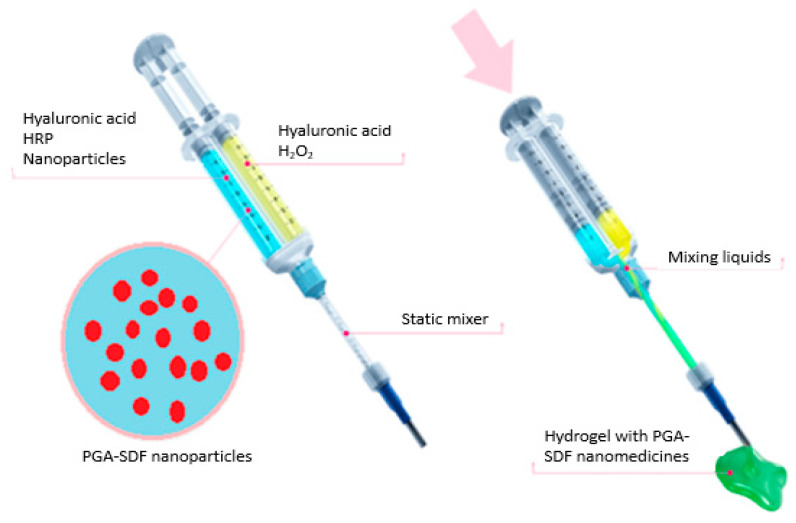
Double syringe injection system used to formulate the star–poly(glutamic acid) (PGA)-Stromal-Derived Factor 1α (SDF) 50:1 nanoparticle-loaded hyaluronic acid hydrogel, showing addition of horseradish peroxidase (HRP) and PGA–SDF nanoparticles to one syringe and hydrogen peroxide (H_2_O_2_) to the other syringe. Adapted with permission from O’Dwyer et al., *Drug Delivery and Translational Research* (2019) [47].

Based on the results of the biocompatibility testing, star-PGA–SDF 50:1 nanoparticles were carried forward as the lead PGA–SDF nanoparticle formulations. These nanoparticles were incorporated into the HA–TA hydrogels. Tyramine-modified hyaluronic acid (HA–TA) (250–350 kDa) with 2–3% tyramine substitution was kindly provided by Contipro (Czech Republic). Hydrogel formation involved dissolving the HA–TA in PBS at the relevant concentration (1% *w/v*), taking account of the volume of nanoparticles to be added. The resulting dispersion was placed on a roller-plate overnight to ensure complete wetting of the freeze-dried HA–TA powder. The HA–TA dispersion was divided into two separate Falcon tubes. The final concentration was adjusted by addition of the star-PGA–SDF 50:1 nanoparticle dispersion to one tube and PBS to the other. Horseradish peroxidase (HRP) was added to one Falcon tube at a concentration of 0.24 U/mL and Hydrogen peroxide (H_2_O_2_) was added to the other tube at 0.88 µmol/mL. H_2_O_2_ causes the oxidation of HRP, which subsequently oxidises the tyramine moieties on the HA. This causes crosslinking due to bond formation between adjacent molecules, either via the ortho carbons of the phenol rings of individual molecules or the ortho carbon of one molecule bound to the phenolic oxygen of another. Dispersions were drawn up into 1 mL syringes and injected through the system shown in Figure 2, which facilitated keeping crosslinkers separate until gelation was required and homogenous mixing of crosslinkers and nanoparticles.

### 2.9. Release of SDF from a Star-PGA–SDF–HA–TA Nano-In-Gel Formulation

The release of SDF from the star-PGA–SDF–HA–TA system was measured in vitro. The star-PGA–SDF–HA–TA was formulated as described in Section 2.8. A 200 µL cylinder of gel, containing 25 ng of SDF as star-PGA–SDF 50:1 nanoparticles, was placed inside a 1 mL cellulose ester membrane Spectra/Por^®^ Float-A-Lyzer^®^ G2 device with a molecular weight cut-off (MWCO) of 300 kDa. 200 µL of PBS was placed on top of the hydrogel sample. The MWCO of the semi-permeable membrane would allow SDF to pass through but would retain intact nanoparticles. 5 mL of release medium (PBS) was placed in the receptor fluid container. The Float-A-Lyzer^®^ container combination was then placed in a 50 mL Falcon tube and put in a water bath at 37 °C shaking at a rate of 75 rpm. The release medium was removed entirely and new, pre-warmed medium was added at each time point. Time points were taken at 8 h and on days 2, 4, 7, 14, 21, 28 and 35. Release medium was frozen at −80 °C for analysis at a later time. Release samples were analyzed via ELISA, which was carried out exactly according to the manufacturer’s instructions. The quantification of SDF was determined by measuring absorbance on a Varioskan plate reader at 450 nm with correction at 570 nm.

### 2.10. Biocompatibility of Star-PGA–SDF–HA–TA

The biocompatibility of the nano-in-gel system containing star-PGA–SDF 50:1 nanoparticles in the HA–TA hydrogel (star-PGA–SDF–HA–TA) was investigated using the release medium from the release study in Section 2.9 as well as release supernatant from a HA–TA hydrogel not loaded with star-PGA–SDF nanoparticles. The release supernatants were pooled separately (star-PGA–SDF–HA–TA or HA–TA), concentrated using an Amicon^®^ Ultra centrifugal filter with a MWCO of 3 kDa, and applied to HUVECs. HUVECs were plated at 3 × 10^4^ cells/well in a 24-well plate. Cells were exposed to the release supernatant for 24 h. Following this, the medium was removed and an MTS assay was carried out exactly as described in Section 2.7.2. The metabolic activity of HUVECs in the release medium was compared to that of cells growing in cell culture medium with no release supernatant present and HUVECs treated with fresh, non-encapsulated SDF at the same concentration as that in the hydrogel release medium, 4.1 ng/mL.

### 2.11. Bioactivity of SDF Released From Star-PGA–SDF–HA–TA

#### 2.11.1. Microvessel Formation—Matrigel^®^ Assay

Bioactivity testing was carried out to investigate if SDF loaded into and released from star-PGA–SDF–HA–TA retained its potency and ability to induce angiogenesis and cell migration. A Matrigel^®^ assay was used to assess the ability of the SDF to induce microvessel formation, indicative of in vivo angiogenic potential. HUVECs were seeded onto 120 µL of growth factor reduced Matrigel^®^ at a seeding density of 3 × 10^4^ in a 48-well plate. The pooled release supernatants from star-PGA–SDF–HA–TA or from HA–TA alone were added to the wells containing HUVEC-seeded Matrigel^®^. Fresh, non-encapsulated SDF, 4.1 ng/mL, was used as bioactivity benchmark control. HUVECs in EndoGrow cell culture medium with no SDF were used as a negative control. The same volume of EndoGrow medium was used in all wells. This medium contained 2% FBS for all groups. Wells were imaged following 6 and 12 h incubation at 37 °C and 5% CO_2_. Five images were taken of each well at 10× magnification and the total tubule length per well was calculated using ImageJ software.

#### 2.11.2. Cell Migration—Scratch Assay

A scratch assay was used to assess the ability of the SDF released from star-PGA–SDF–HA–TA to induce migration. HUVECs were seeded on a 24-well plate at a seeding density of 3 × 10^4^ cells per well and fed with complete endothelial medium until a confluent monolayer of cells was present in the plate. Medium was then removed from all wells and a P200 pipette tip was used to scratch a vertical line down through the cell monolayer thus removing the cells in this location. Each of the wells was washed three times with 500 µL PBS to remove the detached cells. Medium, without FBS, but containing star-PGA–SDF–HA–TA or HA–TA alone supernatants or fresh, non-encapsulated SDF (4.1 ng/mL) was added to the wells. Wells were imaged at 5× magnification at time zero and the position of the image marked on the plate to ensure consistency of imaging throughout the experiment. Images were taken at 0, 6, 12 and 24 h. Gap distance was measured in three locations on the image at each time point using ImageJ software.

#### 2.11.3. Cell Migration—Transwell^®^ Migration Assay

**Figure 3:**
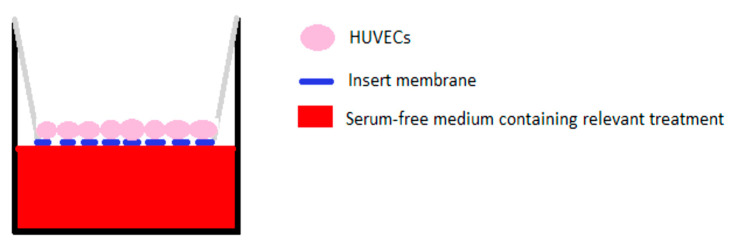
Schematic of a Transwell^®^ migration assay. Human Umbilical Vein Endothelial Cells (HUVECs) were seeded on the top of the semi-permeable membrane with treatments, star-poly(glutamic acid) (PGA) – Stromal-Derived Factor 1α (SDF)–HA–TA supernatant or HA–TA alone supernatant, placed in the bottom of the well, underneath the membrane. In the presence of a migratory stimulus HUVECs will travel through the membrane.

A Transwell^®^ migration assay was performed as a measure of the migration induced by the pooled release supernatant. Corning^®^ Transwell^®^ hanging inserts, with a pore size of 8 µm, large enough to allow passage of HUVECs through the membrane, were placed in wells of a 24-well plate containing 600 µL of serum-free medium per well (Figure 3). HUVECs at P4 or P5 were seeded on the top side of the insert membrane at a density of 3 × 10^4^ cells/insert. The plate was placed in a cell culture incubator for two hours to allow attachment of the cells to the top side of the insert. Following this, treatment and control groups were placed in a fresh 24-well plate. Serum-free medium was used in the control, cells alone, group. Fresh, non-encapsulated SDF or pooled supernatants from star-PGA–SDF–HA–TA or HA–TA alone were added in serum-free medium. The cell-coated inserts were then placed in the wells of the plate containing the control and treatment groups and left in an incubator (37 °C, 5% CO_2_) for 24 h. The plate was then removed from the incubator. The top of each insert was wiped with a cotton bud to remove cells remaining on the top. The bottom of the insert, which had been immersed in the medium and treatment was stained with 200 µL of 2 µM calcein AM. Following 15 min incubation protected from light at room temperature, the bottom of the insert was imaged and two representative images from each insert were taken. The number of cells per image was calculated using the particle counting function on ImageJ.

### 2.12. Statistical Analysis

All statistical tests were performed using GraphPad Prism v5 (GraphPad Software Inc., San Diego, CA, USA). Mean and standard error of the mean are presented on all graphs. A one-way analysis of variance (ANOVA) followed by Bonferroni post-hoc test was used to analyze the data obtained from the Matrigel^®^, scratch and Transwell^®^ migration assays. Significance was determined as *p* < 0.05. Three repeats were performed for all experiments.

## 3. Results

### 3.1. Nanoparticle Fabrication and Physicochemical Characterisation

Formulations consisted of L-PGA–SDF or star-PGA–SDF at one of three molar ratios: PGA:SDF 30:1, PGA:SDF 40:1 or PGA:SDF 50:1. The molar ratios were based on a star-PGA molecular weight of 42 kDa, L-PGA molecular weight of 26 kDa and an SDF molecular weight of 8 kDa. The required amount of star-PGA or L-PGA for each formulation was calculated based on 25 ng SDF in each formulation, the molecular weight of the polypeptides and the molar ratios required.

Results of DLS studies, shown in Table 1, indicated that nano-sized particles were formed at all of the L-PGA:SDF and star-PGA:SDF ratios tested. L-PGA–SDF nanoparticles ranged in size from 255.2 nm to 305.7 nm, while star-PGA–SDF formulations measured between 261.8 nm and 279.4 nm. No clear relationship was evident between PGA:SDF ratio and particle size for either the L-PGA–SDF or the star-PGA–SDF formulations. There was no significant difference in particle size between the L-PGA–SDF and the star-PGA–SDF formulations at equivalent molar ratios or between different molar ratios of the L-PGA–SDF or star-PGA–SDF formulations. The polydispersity index (PDI) of the star-PGA–SDF formulations reduced with increasing molar ratios of star-PGA:SDF, from 0.6 for star-PGA–SDF 30:1 to 0.3 for star-PGA–SDF 50:1. No such correlation was observed for the L-PGA–SDF formulations.

The zeta potential data demonstrated that all L-PGA–SDF and star-PGA–SDF formulations had a negative zeta potential. This was close to neutral for all of the star-PGA–SDF formulations ranging from −3.1 mV to −4.3 mV.

### 3.2. Nanoparticle Tracking Analysis

**Figure 4:**
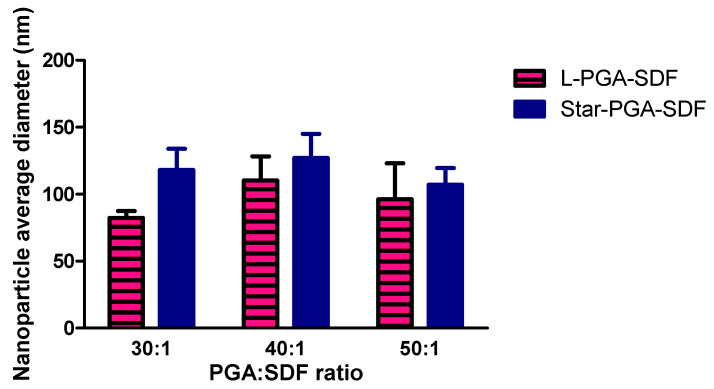
Nanoparticle average diameter of L-poly(glutamic acid) (PGA)—Stromal-Derived Factor 1α (SDF) and star-PGA–SDF formulations at molar ratios of 30:1, 40:1 and 50:1 measured using nanoparticle tracking analysis (*n* = 3). No significant difference between the average diameter of L-PGA–SDF and star-PGA–SDF formulations at any given ratio.

Particle size measurements obtained using Nanoparticle Tracking Analysis are shown in Figure 4. The concentration of particles in all samples was in the range of 10^9^ particles/mL for all L-PGA–SDF and star-PGA–SDF formulations. This work confirmed that, as seen with DLS, L-PGA–SDF and star-PGA–SDF 30:1, 40:1 and 50:1 formulations all formed particles in the nano size range, although the sizes obtained with NTA were smaller than those obtained on DLS. L-PGA–SDF formulations had a smaller average size than their star-PGA–SDF comparator at each individual molar ratio, however, there was no statistically significant difference in particle sizes. No correlation was noted between PGA:SDF molar ratio and particle size. Figure 4 represents the average diameter of three different batches of nanoparticles, showing that batch-to-batch variability in nanoparticle size reduces with an increasing molar ratio for star-PGA–SDF nanoparticles, but increases with increasing molar ratio for L-PGA–SDF nanoparticles.

### 3.3. Transmission Electron Microscopy

**Figure 5:**
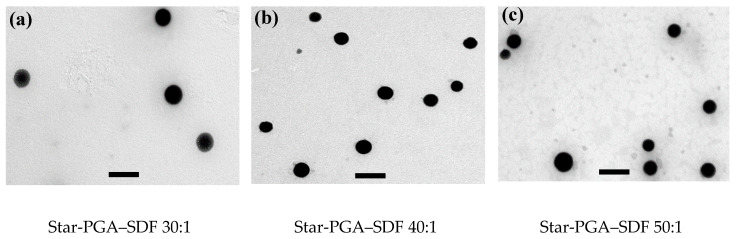
Transmission Electron Microscopy images taken at 26,500× of the (**a**) star-poly(glutamic acid) (PGA) – Stromal-Derived Factor 1α (SDF) 30:1, (**b**) star-PGA–SDF 40:1 and (**c**) star-PGA–SDF 50:1 nanoparticles. No obvious difference in morphology can be observed between the groups. Scale bar = 500 nm.

Representative images of Star-PGA–SDF nanoparticles were acquired using TEM. Imaging confirmed that all three formulations, star-PGA–SDF 30:1, 40:1 and 50:1, formed nanoparticles. As shown in Figure 5, the nanoparticles formed were circular for all formulations and no obvious difference in the nanoparticle morphology was observed between the groups. Nanoparticle size was measured for all three formulations. Star-PGA–SDF 30:1 formulations had an average size of 193 nm (±45 nm), star-PGA–SDF 40:1 measured 143 nm (±43 nm), while star-PGA–SDF 50:1 nanoparticles averaged 182 nm (±53 nm).

### 3.4. Nanoparticle Complexation Efficiency and Loading Content

As shown in Table 2, SDF complexation efficiency for all L-PGA–SDF and star-PGA–SDF formulations was greater than 99.9% *w/w* indicating almost complete complexation of the SDF with either the L-PGA or the star-PGA. Optimisation work on this experiment suggested that 99% of free SDF is eluted through the column. Accounting for this, the complexation efficiency remains greater than 99.9% for all formulations. There were no significant differences in complexation efficiency between the L-PGA–SDF and the star-PGA–SDF formulations. Loading content varied from 0.61% *w/w* to 1% *w/w* for the L-PGA–SDF formulations and 0.38% *w/w* to 0.63% *w/w* for star-PGA–SDF formulations depending on the molar ratio of PGA to SDF used.

### 3.5. Biocompatibility of L-PGA–SDF and Star-PGA–SDF Nanoparticles

**Figure 6:**
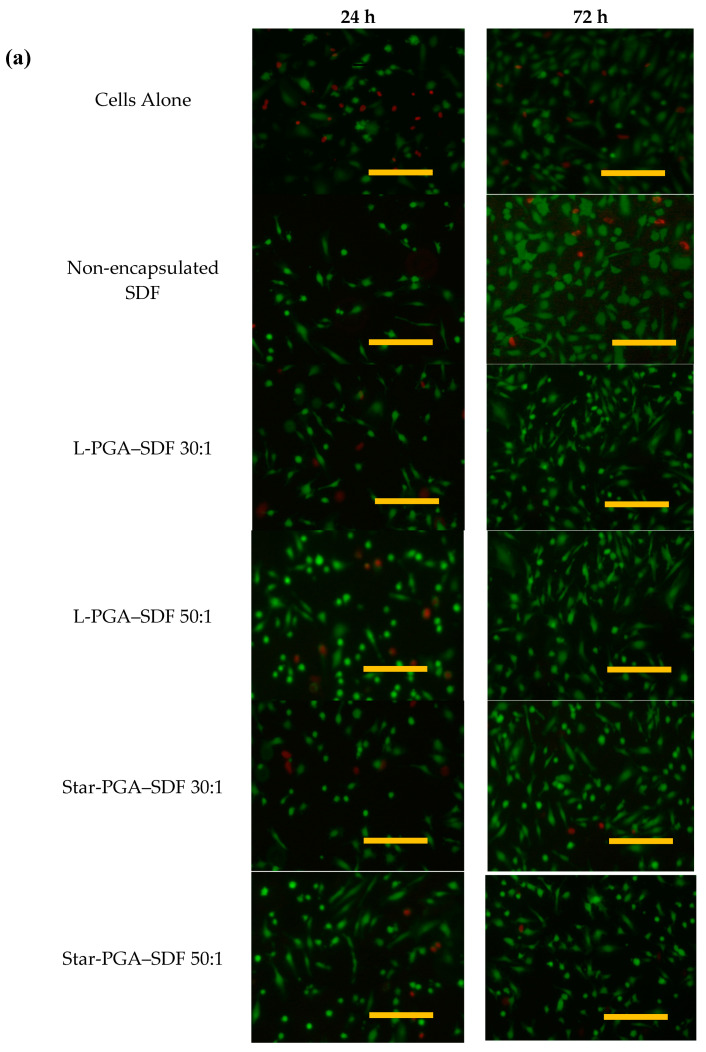

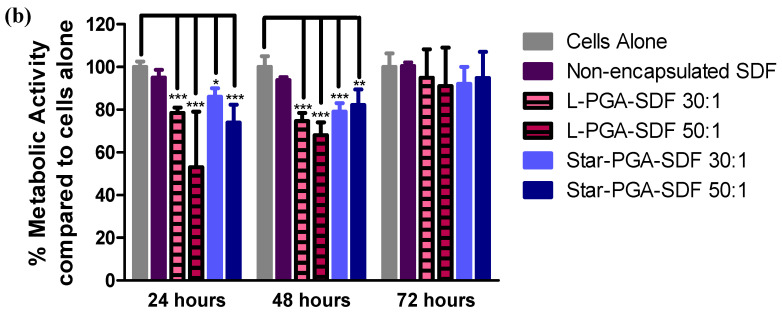
Biocompatibility of L-poly(glutamic acid) (PGA)—Stromal-Derived Factor 1α (SDF) and star-PGA–SDF nanoparticles at PGA:SDF molar ratios of 30:1 and 50:1 containing 25 ng SDF (**a**) Live/Dead images of human umbilical vein endothelial cells (HUVECs) exposed to nanoparticle formulations for 24 or 72 h, Scale bar = 200 µm. (**b**) metabolic activity of HUVECs exposed to the formulations listed for 24, 48 or 72 h measured using a MTS assay (*n* = 3). * *p* < 0.05, ** *p* < 0.01, *** *p* < 0.001, compared to the metabolic activity of cells alone at the specific time point.

Biocompatibility of the nanoparticles was assessed to determine their suitability for biological administration. L-PGA–SDF and star-PGA–SDF 30:1 and 50:1 formulations were carried forward for in vitro testing. The L-PGA–SDF 30:1 and 50:1 and star-PGA–SDF 30:1 and 50:1 formulations were chosen to assess the extreme values of the formulations, i.e., the lowest and highest dose of PGA respectively, as the 30:1 formulations have the least star-PGA or L-PGA and the 50:1 formulations contain the most star-PGA or L-PGA. Figure 6 shows HUVECs exposed to L-PGA–SDF and star-PGA–SDF 30:1 and 50:1 formulations containing 25 ng SDF for 24 or 72 h. In the control groups of cells alone (given complete endothelial medium) and non-encapsulated SDF (given complete endothelial medium supplemented with 25 ng/mL fresh SDF) more cells are present at 72 h than at 24 h, indicating that cell proliferation occurred in these groups. Proliferation between 24 and 72 h is also evident in the images of the L-PGA–SDF 30:1 and 50:1 and star-PGA–SDF 30:1 and 50:1 treatment groups. Figure 6b shows that at both 24 and 48 h exposure of cells to L-PGA–SDF 30:1 and 50:1 and star-PGA–SDF 30:1 and 50:1 led to significantly reduced metabolic activity compared to cells not exposed to these treatments as measured using an MTS assay. At 72 h there is no significant difference in metabolic activity between the treatment or control groups or between the L-PGA–SDF and star-PGA–SDF treated HUVECs indicating that the cells have recovered from the initial cytotoxicity.

### 3.6. Formulation and Characterisation of Star-PGA–SDF–HA–TA

As outlined above, delivery and retention of nanoparticles at the disease site can potentially be facilitated using a nano-in-gel formulation. A lead candidate nanoparticle formulation was selected for loading into a HA–TA hydrogel. L-PGA–SDF 50:1 nanoparticles caused the greatest reduction in metabolic activity at both 24 and 48 h and so were deemed unsuitable for hydrogel loading. Star-PGA–SDF 50:1 nanoparticles had a better PDI than the star-PGA–SDF 30:1 and the L-PGA–SDF 30:1 formulations. Furthermore, in other work by our group star-PGA-VEGF 50:1 nanoparticles have been successfully loaded into a similar HA–TA hydrogel [47]. Thus, the star-PGA–SDF 50:1 nanoparticle formulation was chosen here for integration into the HA–TA hydrogel.

#### 3.6.1. SDF Release from Star-PGA–SDF–HA–TA

Star-PGA–SDF 50:1 nanoparticles were incorporated into a HA–TA hydrogel to give a final concentration of 25 ng SDF per 200 µL hydrogel portion. SDF release from the nano-in-gel system was measured to ensure SDF could be released from the system and that the hydrogel/nanoparticle double matrix system facilitated sustained SDF release. Sustained SDF release was detected up to day 35 with no release detected between day 35 and day 42 (Supplementary Material, Figure S1). In total, 16.5% of the loaded SDF was recovered over the 42 day release study.

#### 3.6.2. Biocompatibility of Star-PGA–SDF–HA–TA

**Figure 7:**
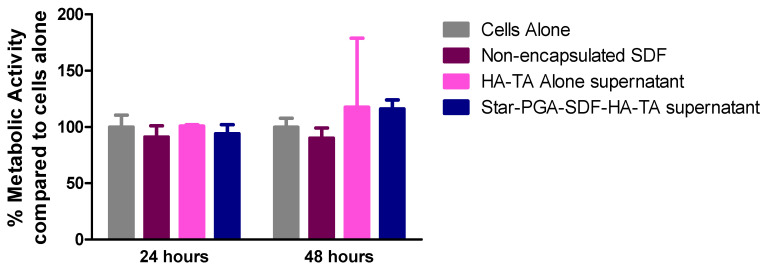
Metabolic activity (MTS assay) of human umbilical vein endothelial cells (HUVECs) exposed to 4.1 ng/mL fresh, non-encapsulated Stromal-Derived Factor 1α (SDF), supernatant from a HA–TA hydrogel or star-poly(glutamic acid) (PGA)–SDF (50:1)-HA–TA supernatant containing 4.1 ng SDF. (*n* = 3).

To assess the biocompatibility of star-PGA–SDF–HA–TA the pooled release medium from the 42-day release study was applied to HUVECs over 24 or 48 h. The control dose of non-encapsulated SDF used, 4.1 ng, matched that present in the hydrogel release supernatant as determined via ELISA. As shown in Figure 7, no significant reduction in metabolic activity was observed on addition of release supernatant from HA–TA alone or star-PGA–SDF–HA–TA to the HUVECs as measured using a MTS assay. No significant differences exist between any of the groups tested indicating the biocompatibility of the star-PGA–SDF–HA–TA supernatant.

### 3.7. Bioactivity of SDF Released from Star-PGA–SDF–HA–TA

SDF is both angiogenic and chemotactic, exerting its effects via interaction with the CXCR7 receptor for angiogenesis and the CXCR4 receptor for cell migration [3,9]. Thorough investigation of the bioactivity of SDF requires assessment of both its ability to induce cell migration and its promotion of angiogenesis. Herein, a Matrigel^®^ assay is used to investiagte angiogenesis via in vitro tubule formation. A scratch assay is used as the initial test of the ability of SDF to induce HUVEC migration following release from star-PGA–SDF–HA–TA. A more difficult test of cell migration, the Transwell^®^ migration assay, is then used to confirm the bioativity of the released SDF.

#### 3.7.1. Microvessel Formation—Matrigel^®^ Assay

**Figure 8:**
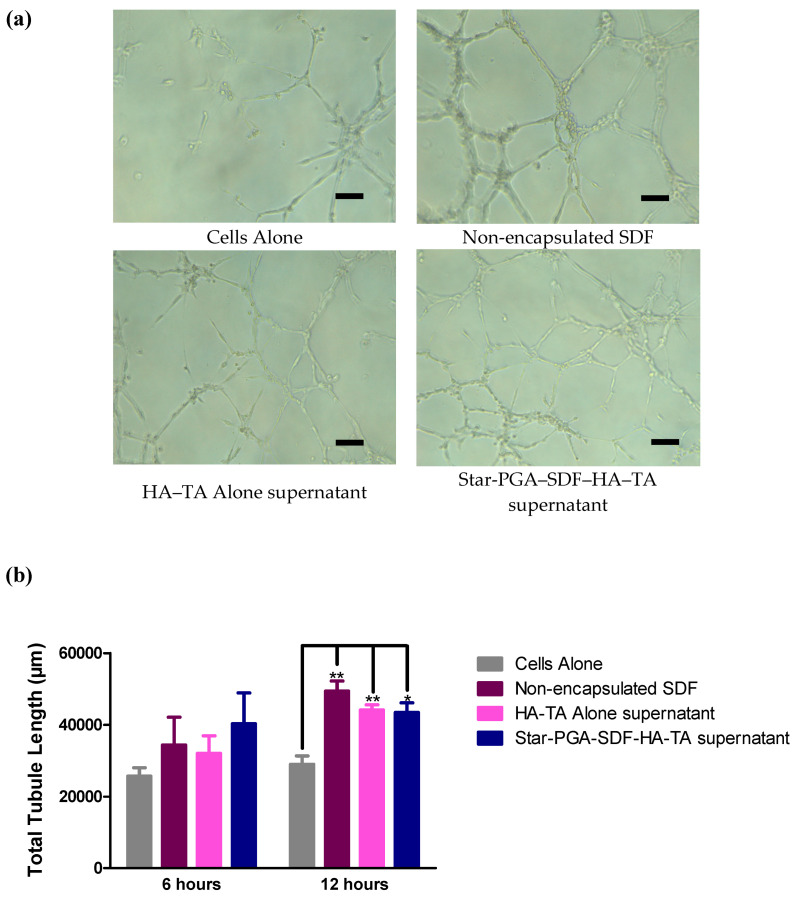
Bioactivity of Stromal-Derived Factor 1α (SDF) released from star-poly(glutamic acid) (PGA)–SDF(50:1)-HA–TA formulation measured on a Matrigel^®^ assay. The total length of the tubule network induced by the released SDF is compared to the network formed by untreated cells (cells alone), fresh, non-encapsulated SDF and supernatant from the hydrogel not loaded with SDF nanoparticles. (**a**) Tubules formed at 12 h in the presence of the treatments listed, showing the lowest amount of microvessel formation in the cells alone group. Scale bar = 100 µm (**b**) Quantification of total tubule length on the Matrigel^®^ assays of samples treated withnon-encapsulated SDF, HA–TA alone supernatant and star-PGA–SDF(50:1)-HA–TA supernatant demonstrated significantly improved total length at 12 h (*n* = 3). SDF concentration in all cases is 4.1 ng/mL. * *p* < 0.05, ** *p* < 0.01.

The release of bioactive SDF from star-PGA–SDF–HA–TA is critical for effective clinical translation. Pooled release medium from HA–TA alone or star-PGA–SDF–HA–TA was applied to HUVECs. Bioactive SDF should induce an increase in the total length of the tubule network formed by the HUVECs. Fresh, non-encapsulated SDF was used as a positive control at the same concentration as that present in the star-PGA–SDF–HA–TA release supernatant, i.e., 4.1 ng SDF/mL of culture medium. The images in Figure 8a and Figure S2 of the Supplementary Material show that the least microvessel formation was observed in the cells alone group, with all other groups promoting more microvessel formation. Quantification of microvessel length, shown in Figure 8b, showed that at 6 h there was no significant difference in total tubule length between any of the groups. However, the average length was lowest in the cells alone group and highest in the group treated with star-PGA–SDF–HA–TA supernatant. At 12 h, 4.1 ng/mL non-encapsulated SDF significantly (*p* < 0.05) increased microvessel length compared to cells alone as did the HA–TA alone and star-PGA–SDF–HA–TA supernatant groups.

#### 3.7.2. Cell Migration—Scratch Assay

**Figure 9:**
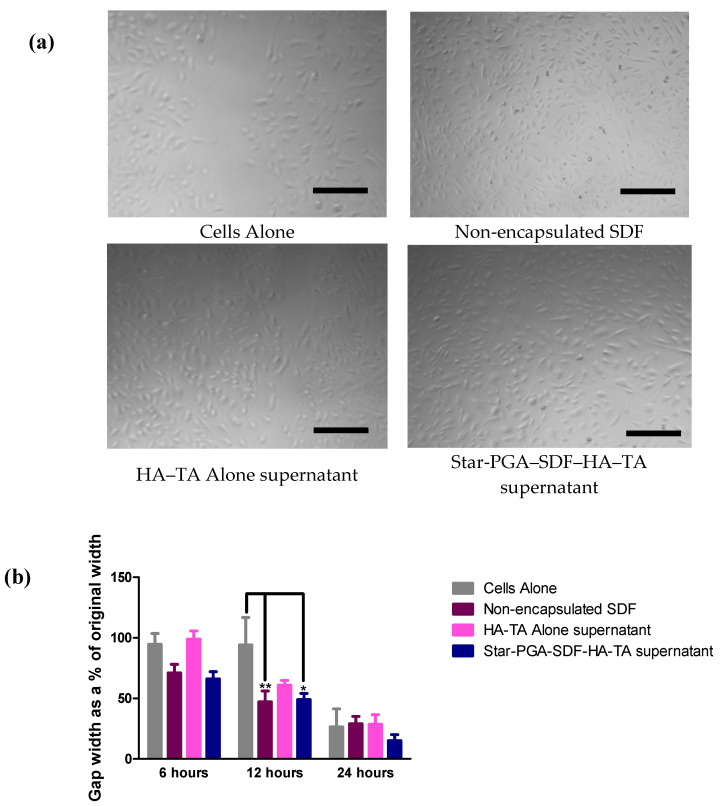
Cell migration assessed via gap closure achieved on a scratch assay. (**a**) Microscopy images of the gap area 12 h following addition of treatments, fresh, non-encapsulated Stromal-Derived Factor 1α (SDF), HA–TA supernatant or SDF released from star-poly(glutamic acid) (PGA)–SDF(50:1)-HA–TA. Scale bar = 100 µm (**b**) Quantification of gap closure showing the best gap closure achieved by star-PGA–SDF(50:1)-HA–TA supernatant. Where SDF is present the concentration is 4.1 ng/mL. (*n* = 3) * *p* < 0.05, ** *p* < 0.01.

SDF is known to promote cell migration in vivo and therefore the efficacy of the SDF formulations was investigated using a scratch assay. Bioactive SDF should promote cell migration closing the formed gap more quickly than untreated cells. Figure 9a shows that, after 12 h, the largest gaps were observed in the cells alone and HA–TA supernatant groups. Better gap closure was observed following 12 h treatment with the non-encapsulated SDF control and the supernatant from the star-PGA–SDF–HA–TA release studies than in the cells alone or HA–TA alone supernatant groups. The quantification of gap closure shown in Figure 9b (as determined using ImageJ software) confirms that, at 12 h, the non-encapsulated SDF and star-PGA–SDF–HA–TA supernatant significantly reduced the gap distance compared to either cells alone or HA–TA alone supernatants. Both non-encapsulated SDF and the equivalent dose of star-PGA–SDF–HA–TA supernatant achieved better gap closure than either cells alone or HA–TA alone supernatant at just 6 h, though the change was not statistically significant for this time point. At 24 h, there were no significant differences between any of the groups, but the star-PGA–SDF–HA–TA supernatant had achieved the best gap closure of any of the groups with only 15% of the original gap width remaining at 24 h. In contrast, 26% of the gap width remained in the cells alone group, with 29% of the original gap width remaining in both the non-encapsulated SDF and HA–TA alone supernatant groups.

#### 3.7.3. Cell Migration–Transwell^®^ Migration

**Figure 10:**
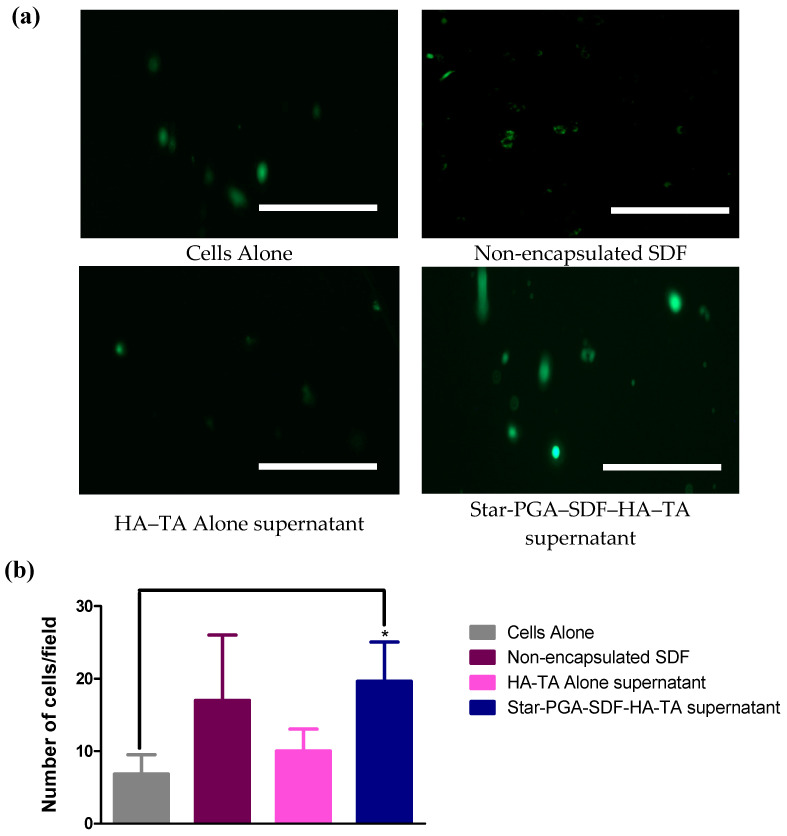
Migration of Human Umbilical Vein Endothelial Cells (HUVECs) through a Transwell^®^ membrane caused by fresh, non-encapsulated Stromal-Derived Factor 1α (SDF), HA–TA alone supernatant or star-poly(glutamic acid) (PGA)–SDF(50:1)-HA–TA supernatant and compared to untreated cells (cells alone) (**a**) Calcein-stained HUVECs imaged after migration through the Transwell^®^ membrane at 24 h Scale bar = 100 µm (**b**) Average number of HUVECs migrated per field—significantly more cells had migrated in the presence of the star-PGA–SDF–HA–TA supernatant than in the untreated cells (cells alone) group. SDF concentration 4.1 ng/mL. (*n* = 3). * *p* < 0.05.

Transwell^®^ migration was also tested to assess if SDF released from the star-PGA–SDF–HA–TA retained its bioactivity and ability to promote cell migration. Bioactive SDF will promote migration of HUVECs through the membrane, resulting in a higher number of cells detected on imaging the underside of the membrane. Figure 10a shows calcein-stained HUVECs, which have migrated through a Transwell^®^ membrane. More cells can be observed in the groups treated with non-encapsulated SDF and star-PGA–SDF–HA–TA supernatant than in the cells alone or HA–TA alone supernatant-treated groups. This is quantified on Figure 10b showing the amount of HUVECs per field which migrated though the Transwell^®^ insert at 24 h. Significantly more HUVECs/field were present in the star-PGA–SDF–HA–TA supernatant-treated group compared to the cells alone group. An average of 19 HUVECs/field had migrated through the membrane in the star-PGA–SDF–HA–TA supernatant group compared to 17 HUVECs/field in the non-encapsulated SDF groups. On average 10 and 6.8 HUVECs/field had migrated in the HA–TA alone supernatant and the cells alone groups, respectively.

## 4. Discussion

SDF is both chemotactic and angiogenic, it can aid in cardiomyocyte recovery following an MI, wound healing and vascularization of tissue engineering scaffolds [12,21,52]. However, its in vivo use is limited by a half-life of just 26 min, making achieving a prolonged effect difficult [4,5,8,26,27]. Herein, we complex the positively charged heparin-binding domain on SDF with negatively charged PGA polypeptides that have either a star-shaped or linear architecture. Differences have previously been reported in the complexation efficiencies, release properties and in vivo half-lives of star-shaped and linear polypeptides. Byrne et al. reported significant differences in nanoparticle size, surface charge and complexation ability between star-shaped and linear poly(lysine) when forming siRNA encapsulating nanoparticles [35]. Duro-Castano et al. reported a three-armed star-PGA with a 13-fold longer in vivo half-life in a mouse compared to L-PGA [53]. Thus, both architecture types, linear and star-shaped, were used here to identify which was the better candidate for SDF nanoparticle formation. The self-assembly of L-PGA–SDF and star-PGA–SDF formulations was achieved after just five minutes of complexation at room temperature. Nanoparticle tracking analysis confirmed the existence of nano-sized particles for all L-PGA–SDF and star-PGA–SDF formulations at ratios of 30:1, 40:1 and 50:1. The nanoparticle sizes obtained via NTA were lower than those identified via DLS. This phenomenon of differing particle sizes between DLS and NTA has previously been reported in the literature and is due to the differing modes of operation of the two instruments [54,55]. When measured using NTA, star-PGA–SDF formulations were 107–127 nm compared to 261.8–279.4 nm on DLS while L-PGA–SDF formulations were 82–110 nm on NTA compared to 255.2–305.7 nm on DLS. These sizes obtained using DLS are similar to previously reported work on controlled release particles for SDF manufactured using PLGA, with PLGA-SDF nanoparticles measuring 255 nm prepared by Dutta et al. [15]. The formation of star-PGA–SDF nanoparticles as well as their size and morphology was further investigated using TEM. TEM of the star-PGA–SDF 30:1, 40:1 and 50:1 formulations confirmed the presence of nanoparticles. The nanoparticle sizes were in the same range as those obtained via DLS and NTA with star-PGA–SDF 30:1, 40:1 and 50:1 measuring 193 nm, 143 nm and 182 nm, respectively. Star-PGA–SDF 30:1, 40:1 and 50:1 nanoparticles were circular and no obvious differences were noted in the nanoparticle morphology between the different molar ratios. The star-PGA–SDF nanoparticles obtained were similar in shape to star–PLL–pDNA nanoparticles previously reported [36].

A slight negative zeta potential was present on all nanoparticles (−3.5 to −6.8 mV for L-PGA–SDF and −3.1 to −4.3 mV for star-PGA–SDF). This negative zeta potential was expected due to the excess of negatively charged L-PGA or star-PGA in the formulations. This surface charge is much closer to neutral than the −23 mV obtained by Bader et al. for their dextran–chitosan–SDF nanoparticles [32]. This neutrality might affect the stability of our PGA–SDF nanoparticles in suspension, causing them to aggregate, but this is less likely to occur when using a hydrogel delivery vehicle. Furthermore, neutral particles are generally less toxic than particles with a large surface charge; thus, the surface charge of these particles might be beneficial for their biocompatibility [56].

Complexation efficiency was almost 100% for all L-PGA–SDF and star-PGA–SDF formulations tested. The complexation efficiency compares favorably to those reported in the literature for PLGA-based SDF nanoparticles of between 61% and 85% [14,15]. Chitosan-based SDF nanoparticles have been reported with complexation efficiencies of 24–90%, all lower than the >99% complexation efficiency we report for L-PGA–SDF and star-PGA–SDF nanoparticles in this paper [12,13,14,15,16]. Yin et al. suggested that using dextran in a nanoparticle formulation would achieve optimal SDF encapsulation because of dextran’s structural similarity to heparin [57]. Yin et al. obtained an encapsulation efficiency of 77–80% for their particles, indicating that the PGA–SDF nanoparticles developed herein, with an encapsulation efficiency of almost 100%, may be better than the current suggested ‘optimal’ system reported in the literature [57]. The protein loading content was higher for L-PGA–SDF formulations than for star-PGA–SDF formulations. The loaded SDF accounted for 1% of the L-PGA–SDF 30:1 formulation and 0.63% of the star-PGA–SDF 30:1 formulation. The loading content reported in this paper for L-PGA–SDF and star-PGA–SDF nanoparticles is 10-fold higher than the SDF loading reported in the literature for PLGA-based SDF nanoparticles [14,15].

To our knowledge, star-shaped or L-PGA have not previously been used to complex with SDF to form nanoparticles. Thus, to assess their potential for biomedical applications early in the development process, in vitro biocompatibility testing was undertaken. L-PGA–SDF 30:1 and 50:1 and star-PGA–SDF 30:1 and 50:1 significantly reduced HUVEC metabolic activity compared to untreated cells at 24 and 48 h. At 72 h, none of the formulations tested, L-PGA–SDF and star-PGA–SDF 30:1 and 50:1, significantly reduced metabolic activity compared to cells alone. This indicates that these novel SDF nanoparticles are suitable for further in vitro testing to assess their bioactivity.

Thus far, we have demonstrated the successful fabrication of a number of PGA–SDF formulations. These formulations are amenable to scale-up due to their potential for self-assembly, provide almost complete SDF complexation and are biocompatible in initial in vitro testing. However, nanoparticles are rapidly cleared from the bloodstream, limiting their potential to have a prolonged effect. Direct injection of a nanoparticle dispersion is therefore not suitable for clinical translation. Incorporation of nanoparticles into a hydrogel delivery vehicle would facilitate delivery and retention at the desired anatomical site. A HA–TA hydrogel was chosen for this purpose. This HA–TA hydrogel is similar to that which has previously shown the ability to encapsulate and release the protein alpha-amylase [43]. A HA hydrogel like the one used herein has also been injected through a percutaneous catheter following MI in a porcine model [46]. Thus, this HA–TA hydrogel represents a good initial delivery vehicle for these SDF nanoparticles, which could have applications in cardiac regeneration. Based on the physicochemical and biocompatibility testing star-PGA–SDF 50:1 nanoparticles were selected as lead nanoparticles andloaded into the HA–TA hydrogels.

Release of SDF from star-PGA–SDF–HA–TA was sustained for up to 35 days in vitro. Although this SDF release period is not as long as the 60-day SDF release obtained in vitro by Dutta and colleagues for their PLGA-based SDF nanoparticles in a fibrin hydrogel, it is longer than the minimum 14-day SDF release required for neural applications [15]. The overall recovery of SDF was low at 16.5%. Such a low recovery rate for released SDF has been previously reported for other drug delivery systems in the literature. Zamproni et al. recovered just 25% of the loaded SDF from their PLGA-based SDF nanoparticles in vitro over 2 weeks, while Bader and colleagues did not recover any SDF from their SDF–dextran–chitosan nanoparticles over seven days and Cross and colleagues reported between 2% and 40% SDF release from their PLGA microspheres over 64 days [14,32,58]. Regarding SDF release from hydrogels in vitro, He et al. recovered 35% of the SDF loaded into poly(lactide ethylene oxide fumarate) hydrogels over 21 days, while Rabbany et al. recovered 60% of the SDF loaded into an alginate hydrogel patch over 150 h and Zhu et al. recovered 27% of the SDF loaded into a poly(polyethylene glycol citrate-*co*-N-isopropylacrylamide) hydrogel over 21 days [59,60,61]. The low recovery of SDF obtained herein and in the literature may be due to degradation of the SDF between time points on the release study or to the heparin-binding site of the SDF remaining attached to the star-PGA. Degradation of SDF between time points was previously reported by He et al. in a 21-day release study in vitro where HPLC analysis of samples showed peaks potentially due to degradation products [59]. In the work of He et al., release samples were taken every four days. Herein, samples were taken every seven days, which can further account for the low recovery of SDF recorded. However, in vivo, SDF can have an effect immediately upon being released and therefore more active SDF could potentially be made available in vivo than is predicted by this in vitro release model.

Another possibility is that the SDF remains attached, via its heparin-binding site, to the star-PGA and is therefore not detected on the ELISA. This phenomenon was observed by Bader et al. whose SDF–dextran–chitosan nanoparticles showed no release of SDF over seven days in vitro. This was suggested to be due to the high affinity of the SDF heparin-binding site for the negatively charged dextran–chitosan nanoparticle carrier [32]. However, Bader and colleagues determined that, despite SDF not being released, the SDF was capable of having a biological effect on a migration assay in vitro, signaling that SDF could exert an effect while still attached to the nanoparticle carrier [32]. In this work, we measured only SDF completely released from both the HA–TA hydrogel and the star-PGA–SDF nanoparticle, but, based on the work by Bader et al., SDF retained in star-PGA–SDF nanoparticles released from star-PGA–SDF–HA–TA may also be capable of exerting an effect.

Degradation products of the star-PGA–SDF–HA–TA nano-in-gel system could potentially have a deleterious effect on cell viability. Therefore, HUVEC metabolic activity in the presence of the hydrogel release supernatant was measured. No significant reduction in metabolic activity was noted after exposure to the star-PGA–SDF–HA–TA supernatant (Figure 7). A similar HA–TA hydrogel did not adversely affect the metabolic activity of adipose-derived stem cells [46] and PGA polypeptides have previously been shown to be biocompatible with HUVECs in vitro at PGA concentrations up to 3 mg/mL [53]. In Figure 6, the star-PGA–SDF nanoparticles alone induced significant reductions in HUVEC metabolic activity at 24 h and 48 h. The difference seen between the effect of the nanoparticles on metabolic activity and the effect of the hydrogel release supernatant on metabolic activity (Figure 7) may be due to the different presentation of the materials to the cells, i.e., nanoparticles versus release supernatant. Although this has not been confirmed experimentally, HA molecules released during hydrogel degradation could be present in the release supernatant and be exerting a protective effect on the cells.

The stability of proteins in sustained release systems has previously been questioned [62]. Herein we determined the bioactivity of the released SDF using three different in vitro tests of SDF bioactivity integral to its physiological actions. The Matrigel^®^ assay indicates a molecule’s ability to increase tubule formation, an essential process in angiogenesis. SDF can have an effect on tubule formation via interaction with the CXCR7 receptor and subsequent activation of tip cells via the PI3K/Akt signalling pathway [9,63]. This CXCR7 receptor is present on HUVECs which accounts for the increases in total tubule length seen here when HUVECs were treated with fresh, free SDF or SDF–containing release supernatant from the star-PGA–SDF–HA–TA system. At 6 h, although there were no significant differences between any of the groups, SDF released from the star-PGA–SDF–HA–TA system produced the greatest total tubule length at 40,279 µm compared to 25,676 µm for cells alone. SDF from the star-PGA–SDF–HA–TA also produced a greater increase in tubule length than either of its component parts with non-encapsulated SDF producing 34,375 µm and HA–TA alone producing 32,004 µm in total tubule length, compared to the 40,279 µm in total tubule length in the star-PGA–SDF–HA–TA supernatant group. At 12 h, non-encapsulated SDF significantly increased the total tubule length compared to cells alone (*p* < 0.01). This total tubule length produced by non-encapsulated SDF at 12 h, 49,351 µm, is similar to that produced by the supernatant from the star-PGA–SDF–HA–TA system of 43,443 µm, although the statistical significance of the increase induced by the SDF released from star-PGA–SDF–HA–TA (*p* < 0.05) is less than that induced by fresh, non-encapsulated SDF (*p* < 0.01). Zhang et al. reported a 1.75-fold increase in tubule formation compared to cells alone on a Matrigel^®^ assay when using a SDF concentration of 20 ng/mL [9]. Herein we have observed, at 12 h, a 1.5-fold increase in total tubule length with 4.1 ng/mL SDF. This indicates the integrity of the SDF is retained on release from the nano-in-gel system. The supernatant from HA–TA gel alone also significantly (*p* < 0.01) increased the total tubule length compared to untreated cells at 12 h on the Matrigel^®^ assay. HA is known to induce angiogenesis, and this emphasises the advantage of this formulation for biomedical applications where angiogenesis is required. There is no significant difference between the total tubule length produced by star-PGA-HA–TA and that produced by HA–TA alone. Increasing the concentration of SDF in the hydrogel in future studies might result in a significant difference between these two groups. Furthermore, SDF is also chemotactic and so its incorporation may lead to in vivo benefits over the HA–TA alone.

A scratch assay was used as the first test of the chemotactic ability of the released SDF. At 6 h, the SDF released from the star-PGA–SDF–HA–TA system had produced the best gap closure with only 66% of the original gap remaining compared to 94.58% remaining in the cells alone group and 71% and 98.9% in the non-encapsulated SDF and HA–TA alone groups. At 12 h, both non-encapsulated SDF and the supernatant from the star-PGA–SDF–HA–TA system had significantly reduced the gap distance (*p* < 0.01 and *p* < 0.05 respectively) compared to cells alone. At 24 h, the smallest gap was seen in the group treated with SDF from star-PGA–SDF–HA–TA, with only 15% of the original gap remaining; this compares to 26.5%, 29% and 28.6% of the original gap remaining in the cells alone, non-encapsulated SDF and HA–TA alone groups, respectively. This, again, indicates that the bioactivity of the SDF is retained over the 35-day release period. SDF released from star-PGA–SDF–HA–TA performed better than fresh, non-encapsulated SDF at the same concentration (4.1 ng/mL), with a 1.2-fold improved gap closure. This is potentially due to molecules other than the SDF in the star-PGA–SDF–HA–TA release supernatant, including HA degradation products or PGA, although the presence and concentrations of these in the supernatant have not been determined. Latifi-Pupovci observed a 30–35% gap closure with mesenchymal stromal cells (MSCs) in vitro after 12 h of exposure to 20 ng/mL SDF [64]. The results presented herein for gap closure at 12 h compare favorably to this, with 53% closure in the presence of 4.1 ng/mL fresh SDF and 51% gap closure in the group exposed to 4.1 ng/mL SDF released from star-PGA–SDF–HA–TA, albeit on HUVECs instead of MSCs.

Finally, a Transwell^®^ migration assay was used as a more challenging test of the chemotactic potential of the released SDF. In this assay, only the supernatant from the star-PGA–SDF–HA–TA-treated group led to a statistically significant improvement in migration compared to cells alone at 24 h (*p* < 0.05). Star-PGA–SDF–HA–TA supernatant induced the migration of 2.9 times more HUVECs than cells alone. The average number of migrated HUVECs in the group treated with fresh, non-encapsulated SDF was 17 compared to 19.7 migrated HUVECs in the star-PGA–SDF–HA–TA supernatant-treated group. This reaffirms that the bioactivity of the SDF was retained over the 35-day release study. The results of this Transwell^®^ migration assay correlate well with those obtained by Zhang et al. where 20 ng/mL SDF doubled the number of cells migrating compared to cells alone at 24 h. Interestingly, the star-PGA–SDF–HA–TA supernatant herein has a lower concentration of SDF, just 4.1 ng.ml, than that used by Zhang et al., but produces a 2.9-fold increase in HUVEC migration [9].

## 5. Conclusions

In this study, we report the complexation of SDF, for the first time, to our knowledge, with both star and linear PGA to form nanoparticles. These PGA–SDF nanoparticles self-assemble with almost complete SDF complexation. The star-PGA–SDF 50:1 nanoparticles were not toxic in the in vitro biocompatibility studies performed and were successfully incorporated into a hyaluronic acid hydrogel similar to those previously investigated for their translational potential in pre-clinical in vivo models. SDF was released from the hydrogel for up to 35 days and relevant in vitro bioactivity assays indicated that the released SDF retained its bioactivity. Thus, we report a nano-in-gel system which provides the sustained release of bioactive SDF in vitro and may be useful for exploiting the positive biological effects of SDF in vivo. Although, in this paper, we have investigated the incorporation of PGA–SDF nanoparticles into a HA–TA hydrogel, these nanoparticles could potentially be used alone or with other drug delivery vehicles or tissue engineering scaffolds for a range of biomedical applications.

## 6. Patents

Some of the work contained herein is covered under patent application number 1821014.6.

## Figures and Tables

**Table 1:** Z-average size, polydispersity index (PDI) and zeta potential of (**a**) Linear-poly(glutamic acid) (PGA)—Stromal-Derived Factor 1α (SDF) formulations at molar ratios of 30:1, 40:1 and 50:1 and (**b**) star-PGA–SDF formulations (*n* = 3).

**(a)**	**L-PGA:SDF Ratio**	**Z-Average Size (nm)**	**PDI**	**Zeta Potential (mV)**
	30:1	275.0	0.6	−6.8
	40:1	305.7	0.5	−3.8
	50:1	255.2	0.6	−3.5
**(b)**	**Star-PGA:SDF Ratio**	**Z-Average Size (nm)**	**PDI**	**Zeta Potential (mV)**
	30:1	279.4	0.6	−3.1
	40:1	261.8	0.6	−4.3
	50:1	275.8	0.3	−4.0

**Table 2:** Complexation efficiency % *w/w* and protein loading content % *w/w* of L-poly(glutamic acid) (PGA)—Stromal-Derived Factor 1α (SDF) and star-PGA–SDF formulations at PGA:SDF molar ratios of 30:1, 40:1 and 50:1 (*n* = 3).

PGA:SDF Ratio	Complexation Efficiency		Protein Loading	
	L-PGA–SDF	Star-PGA–SDF	L-PGA–SDF	Star-PGA–SDF
30:1	99.97	100	1	0.63
40:1	99.96	99.98	0.76	0.47
50:1	99.96	99.98	0.61	0.38

## Supplementary Materials

The following are available online at https://www.mdpi.com/1999-4923/12/6/513/s1. Figure S1. (a) Release of SDF from the star-PGA-SDF(50:1)-HA-TA nano-in-gel system (25 ng SDF/200 µL hydrogel portion) over 42 days (*n* = 3) showing sustained SDF release for up to 35 days with a plateau thereafter. Figure S2. Microvessel formation at 12 h on a Matrigel^®^ assay to determine the bioactivity of SDF released from star-PGA-SDF(50:1)-HA-TA. The total tubule length induced by the released SDF is compared to the network formed by untreated cells, fresh, non-encapsulated SDF (4.1 ng/mL) and supernatant from the hydrogel not loaded with SDF nanoparticles. (*n* = 3). * *p* < 0.05, ** *p* < 0.01. Scale bar = 100 µm.
