# Supplementary material for: Development of a Sustained Release Nano-In-Gel Delivery System for the Chemotactic and Angiogenic Growth Factor Stromal-Derived Factor 1α
Source: Pharmaceutics. 2020 Jun 4;12(6):513. doi: 10.3390/pharmaceutics12060513 (PMC7355599; doi:10.3390/pharmaceutics12060513)
Supplement: Supplementary file 1 [file pharmaceutics-12-00513-s001.pdf]

pharmaceutics

MDPI

Supplementary Materials: Development of a Sustained Release Nano-In-Gel Delivery System for the Chemotactic and Angiogenic Growth Factor Stromal-Derived Factor 1α

Joanne O'Dwyer, Megan Cullen, Sarinj Fattah, Robert Murphy, Smiljana Stefanovic, Lenka Kovarova, Martin Pravda, Vladimir Velebny, Andreas Heise, Garry P. Duffy and Sally Ann Cryan

Release of SDF from star-PGA-SDF(50:1)-HA-TA

SDF release from the HA-TA hydrogel loaded with star-PGA-SDF nanoparticles (star-PGA-SDF-HA-TA) was measured. Each 200 µl hydrogel contained 25 ng SDF. Sustained SDF release was detected up to day 35 with no release detected between day 35 and day 42 (Figure S1). 16.5% of the total loaded SDF was recovered over the 42 day release study. At the end of the study the hydrogels were degraded with hyaluronidase and the resulting liquid was assayed for SDF us*ing ELISA. No SDF was detected.*

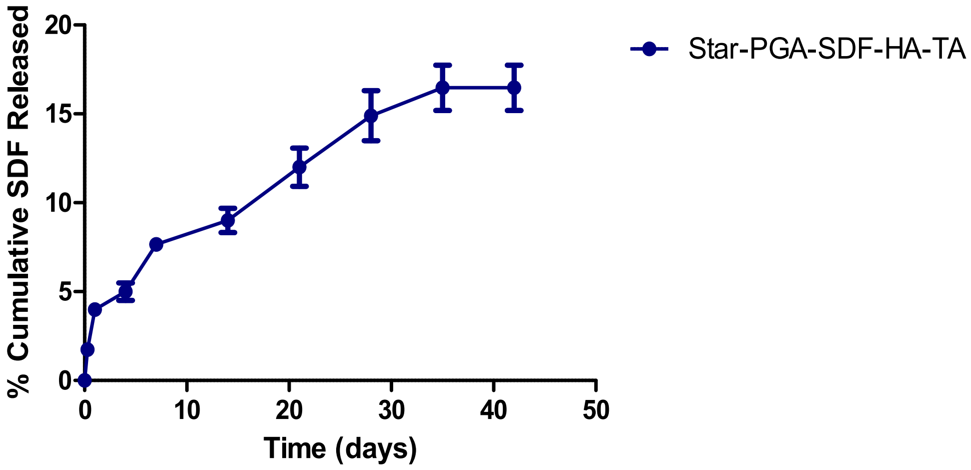

Figure S1. (a) Release of SDF from the star-PGA-SDF(50:1)-HA-TA nano-in-gel system (25 ng SDF/200 µl hydrogel portion) over 42 days (n=3) showing sustained SDF release for up to 35 days with a plateau thereafter.

Microvessel formation - Matrigel® assay additional images

A Matrigel® assay was used to determine the bioactivity of the SDF released from star-PGA-SDF(50:1)-HA-TA. The pooled release supernatant was applied to HUVECs seeded on Matrigel®. Five images of each well were taken at each time point and the total tubule length for each well was measured using ImageJ software. The quantification of the total tubule length is shown in Figure 8 (b) of the manuscript. An additional panel of images from the Matrigel® assay is shown in Figure S2 to exemplify the differences between the cells alone negative control group and the other three groups, non-encapsulated SDF (4.1 ng/ml), HA-TA Alone supernatant and star-PGA-SDF-HA-TA supernatant.

	**Image 1**	**Image 2**	**Image 3**
**Cells Alone**	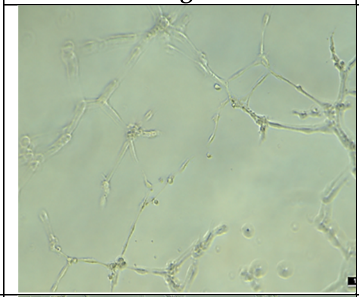	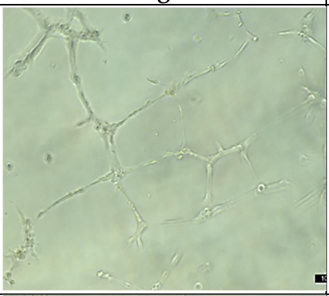	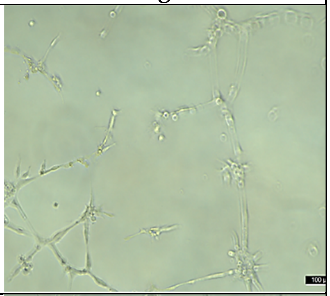
**Non-encapsulated SDF**	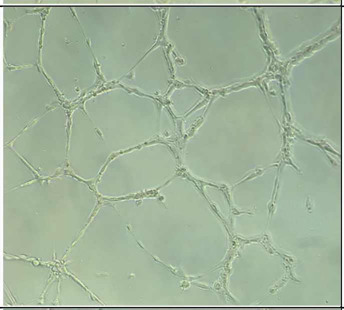	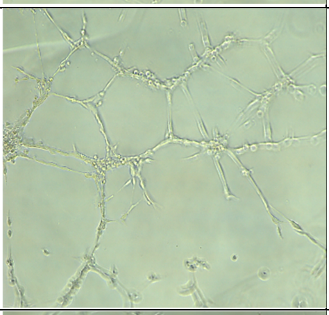	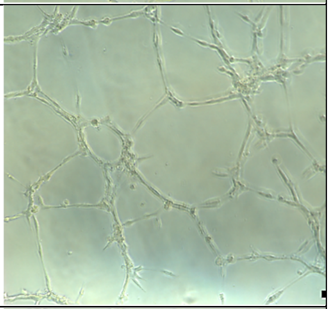
**HA-TA Alone supernatant**	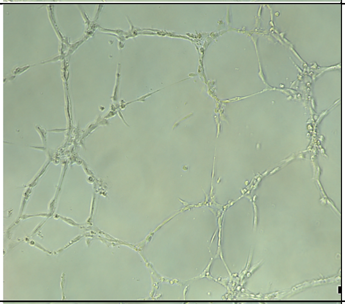	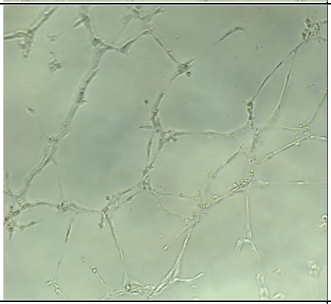	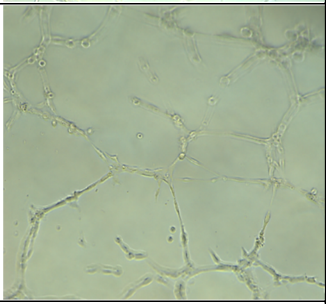
**Star-PGA-SDF-HA-TA supernatant**	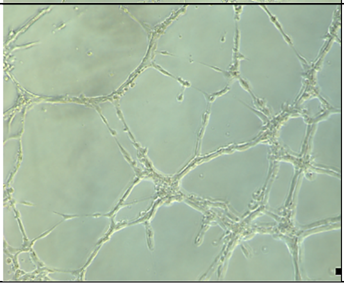	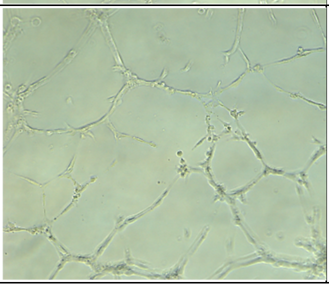	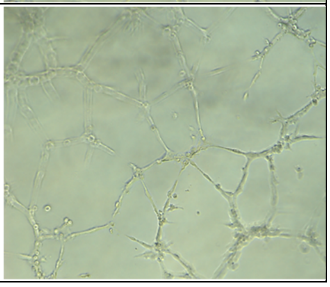

Figure S2. Microvessel formation at 12 hours on a Matrigel® assay to determine the bioactivity of SDF released from star-PGA-SDF(50:1)-HA-TA. The total tubule length induced by the released SDF is compared to the network formed by untreated cells, fresh, non-encapsulated SDF (4.1 ng/ml) and supernatant from the hydrogel not loaded with SDF nanoparticles. (n = 3). *$p < 0.05$, **$p < 0.01$. Scale bar = 100 μm.
